# A neutralizing single-domain antibody that targets the trimer interface of the human metapneumovirus fusion protein

**DOI:** 10.1128/mbio.02122-23

**Published:** 2023-12-20

**Authors:** Marlies Ballegeer, Revina C. van Scherpenzeel, Teresa Delgado, Maria Iglesias-Caballero, Blanca García Barreno, Shubham Pandey, Scott A. Rush, Joost A. Kolkman, Vicente Mas, Jason S. McLellan, Xavier Saelens

**Affiliations:** 1 VIB Center for Medical Biotechnology, VIB, Ghent, Belgium; 2 Department of Biochemistry and Microbiology, Ghent University, Ghent, Belgium; 3 Janssen Infectious Diseases and Vaccines, Beerse, Belgium; 4 Centro Nacional de Microbiología, Instituto de Salud Carlos III, Madrid, Spain; 5 Department of Molecular Biosciences, The University of Texas at Austin, Austin, Texas, USA; Icahn School of Medicine at Mount Sinai, New York, New York, USA

**Keywords:** human metapneumovirus, single-domain antibody, fusion protein, structure

## Abstract

Human metapneumovirus (hMPV) is a leading cause of viral lower respiratory tract disease in children and adults. The hMPV fusion protein F is a trimeric class I fusion protein that is initially synthesized as a precursor (F_0_) and requires proteolytic activation by a host cell protease to generate the metastable, fusion-competent prefusion conformation of F. hMPV F is considered the main target of the neutralizing antibody response against hMPV infection. We isolated single-domain antibodies (sdAbs) directed against hMPV F that potently neutralize hMPV A and B strains. One of these sdAbs, sdHMPV16, specifically bound to cleaved and uncleaved prefusion F. Co-crystal structure analysis revealed that sdHMPV16 binds to a site located at the trimer interface of prefusion F. Moreover, prophylactic treatment with a sdHMPV16-Fc fusion protein reduced viral titers in the lungs of hMPV-infected cotton rats. In summary, sdHMPV16 broadly neutralizes hMPV, can be turned into a candidate biologic that restricts hMPV replication in an *in vivo* model, and, unexpectedly, binds to an unconventional epitope at the prefusion F trimer interface.

**IMPORTANCE:**

Human metapneumovirus (hMPV) is an important respiratory pathogen for which no licensed antivirals or vaccines exist. Single-domain antibodies represent promising antiviral biologics that can be easily produced and formatted. We describe the isolation and detailed characterization of two hMPV-neutralizing single-domain antibodies that are directed against the fusion protein F. One of these single-domain antibodies broadly neutralizes hMPV A and B strains, can prevent proteolytic maturation of F, and binds to an epitope in the F trimer interface. This suggests that hMPV pre-F undergoes trimer opening or “breathing” on infectious virions, exposing a vulnerable site for neutralizing antibodies. Finally, we show that this single-domain antibody, fused to a human IgG1 Fc, can protect cotton rats against hMPV replication, an important finding for potential future clinical applications.

## INTRODUCTION

Human metapneumovirus (hMPV) was first reported in 2001 and is a leading cause of acute respiratory tract infections in children, immunosuppressed patients, and the elderly (1–3). It is estimated that up to 86% of infants under 5 years are affected by this virus (4). With an estimated 14.2 million hMPV-associated acute lower respiratory tract infection cases in children younger than 5 years, the health and economic impact due to hMPV is significant (3, 5, 6). There are no clinically approved vaccines or antivirals to prevent or treat disease caused by hMPV infection. hMPV isolates cluster in two antigenically distinct lineages, named A and B, which are further divided into four lineages: A1, A2, B1, and B2 (7). The hMPV genome encodes three membrane proteins: the small hydrophobic (SH), the attachment (G), and the fusion (F) protein. hMPV F is highly conserved among hMPV sublineages and is indispensable for hMPV infection (8–10). hMPV F is a class I fusion protein that structurally resembles the F protein of respiratory syncytial virus (RSV). F of these two viruses exists in at least two distinct conformations, the prefusion (Pre-F) and the post-fusion (Post-F) state (11, 12). hMPV Pre-F is derived from its precursor F_0_ by cleavage at a single site to generate a homotrimer composed of three disulfide-linked F_1_-F_2_ protomers. F_0_ can be cleaved by transmembrane proteases such as TMPRSS22 at the cell surface or once incorporated into the virus particle (13). In *in vitro* assays, trypsin can be used to convert F_0_ into fusion-competent Pre-F (14). F_0_ cleavage liberates the fusion peptide, which is buried inside a hydrophobic cavity where it interacts with adjacent protomers leading to the stabilization of the trimeric conformation (11, 15). Pre-F undergoes major conformational rearrangements to the Post-F state during membrane fusion, a process that starts with the insertion of the hydrophobic fusion peptide, positioned at the N-terminus of F_1_, into the target cell membrane (16). Crystal structures of trimeric hMPV Pre- and Post-F have been resolved for the hMPV A1 subgroup (11, 17). To express, purify, and crystallize hMPV F in its prefusion state, soluble, recombinant F was stabilized with a proline substitution to prevent its refolding to the Post-F conformation and was fused at the C-terminus of its ectodomain to a trimerizing foldon domain. More recently, cavity filling, other stabilizing substitutions, and the incorporation of disulfide bridges have been combinatorially applied to generate highly expressed, stable Pre-F derived from hMPV A1 (18).

Neutralizing antibodies elicited by hMPV infection are primarily directed against F (10). Many of the isolated human monoclonal antibodies directed against hMPV F bind to both Pre-F and Post-F conformations, whereas binding to Pre-F is required for neutralization (11, 19). Nevertheless, a substantial percentage of hMPV Pre-F-specific human monoclonal antibodies isolated from two human donors have only weak neutralizing activity although they bind Pre-F with high affinity, underlining the complexity of the native hMPV F trimer and the neutralizing antibody response (20). Several antigenic sites on the surface of hMPV F have been identified including antigenic sites II, III, and IV (reviewed in reference (12)) and one targeted by mAb DS7 (21). A novel epitope present at the Pre-F trimer interface that includes the 66–87 alpha helix targeted by mAb MPV458 was recently reported (22). Unlike for RSV F, immune dominance of hMPV Pre-F specific antigenic site Ø and V at the top of Pre-F has not been described, likely because the apex of hMPV Pre-F is covered by a glycan shield and thus less accessible for antibody recognition (11). However, a systematic interrogation of the epitopes of more than 100 human monoclonal antibodies revealed broad recognition of the F protein surface (23).

Next to conventional antibodies, several species such as camelids and nurse sharks also produce heavy chain-only antibodies (HCAbs), which implies that their antigen-binding domain can be expressed as a single-domain antibody (sdAb also named VHH or nanobody). High-affinity sdAbs against a broad spectrum of viral antigens have been described (24). For RSV, for example, three Pre-F conformation-specific sdAbs, that target a distinct epitope in RSV pre-F, have been reported (25–27). Because of their extended complementarity-determining region 3 (CDR3) and small size (approximately 15 kDa), sdAbs can target unique antigenic sites that are difficult to access by conventional antibodies (28, 29). For example, Xun et al. described a sdAb that binds to epitope VI on RSV F protein, which is located very close to the membrane (27).

Here, we describe the isolation and characterization of a llama-derived sdAb (sdHMPV16) that neutralizes hMPV A and B strains and selectively binds to hMPV F in the prefusion conformation. Structural studies reveal that sdHMPV16 binds to an epitope in Pre-F that is located at the trimer interface between the protomers within the hMPV trimer. We also demonstrate that sdHMPV16 fused to the Fc domain of a conventional human IgG1 reduces hMPV replication in cotton rats when administered prophylactically.

## RESULTS

### Isolation of sdAbs that target hMPV F

**Fig 1:**
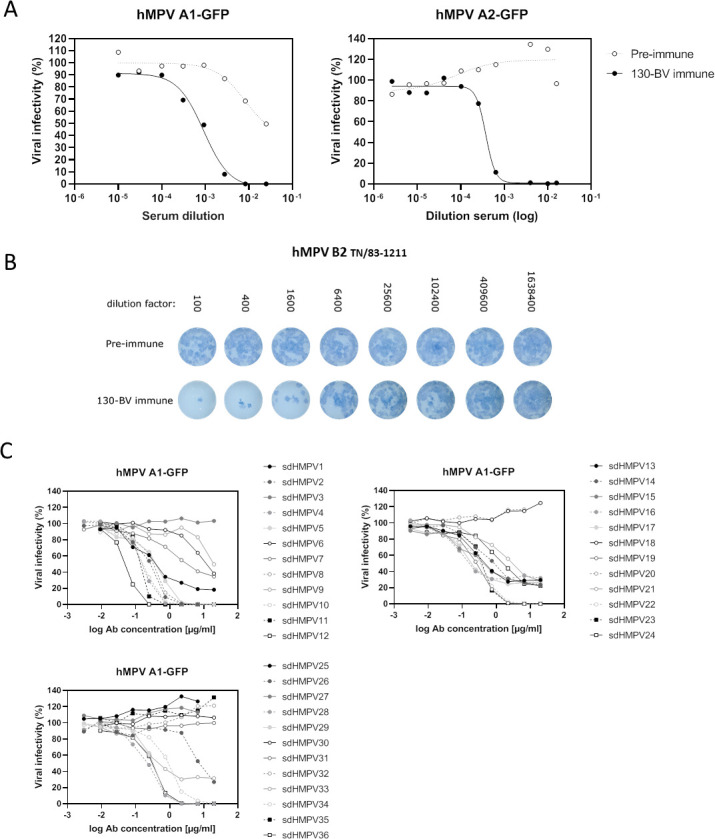
Llama immunization with Pre-F hMPV F induces hMPV neutralizing antibodies. (**A**) hMPV A1-GFP isolate NL/01/00 (left panel) or hMPV A2-GFP isolate CAN97-83 (right panel) was pre-incubated with different dilutions of pre-immune or 130-BV immune serum before inoculation of Vero118 cells or LLC-MK2 cells, respectively. Twenty-four to 72 hours later, the GFP fluorescence was measured and fluorescence intensities were expressed as a percentage of a virus control without sdAb (% viral infectivity). (**B**) hMPV B2 (TN/83–1211) virus was pre-incubated with different dilutions of pre-immune or 130-BV immune serum before infection of LLC-MK2 cells overlayed with 0.3% avicel. After 5 days, the viral plaques were stained with mouse anti-hMPV serum. (**C**) hMPV A1-GFP (NL/1/00) was pre-incubated with different concentrations of 36 selected sdAb candidates (sdHMPV1-36) before inoculation of Vero118 cells. Twenty-four to 48 hours later, the GFP fluorescence was measured and fluorescence intensities were expressed as a percentage of a virus control without sdAb (% viral infectivity). The results are depicted on three different graphs to make them more clear.

To generate hMPV F-specific sdAbs, a llama was immunized with the 130-BV immunogen, a recombinant, uncleaved F protein derived from the hMPV A1 strain with antigenic characteristics of the Pre-F conformation (11). This immunogen was generated by the combination of a single proline substitution (A185P) and a C-terminal trimerization motif (foldon) appended to the hMPV F ectodomain (30). Blood samples were collected before (pre-immune) and after six weekly immunizations (130-BV immune) and serum was prepared. The 130-BV immune serum could neutralize hMPV A1 (NL/1/00)- and A2 (CAN97-83)-GFP reporter viruses as well as a hMPV B2 (TN/83–1211) strain (Fig. 1A and B). Thus, immunization of a llama with 130-BV induced a broad hMPV-neutralizing serum antibody response. Next, peripheral blood mononuclear cells were isolated from a blood sample taken on day 5 after the last immunization to construct a sdAb phage display library, which was used for bio-panning on immobilized 130-BV. Candidate hMPV-neutralizing sdAbs were identified by randomly selecting clones obtained after panning. These clones were grown individually for small-scale production of a periplasmic extract (PE) which was then tested for binding to 130-BV in ELISA (data not shown). All clones with a 130-BV to BSA background signal ratio higher than 2 were sequenced and unique 130-BV-binding clones were selected for further characterization. Based on the PE ELISA and sequencing results, the cDNA inserts of 36 unique sdAb candidates were cloned into a *P. pastoris* expression vector. The resulting 36 sdAbs were purified from the yeast culture medium and tested for their hMPV A1 (NL/1/00)-neutralizing activity (Fig. 1C).

### sdHMPV16 neutralizes both hMPV A and B strains

**Fig 2:**
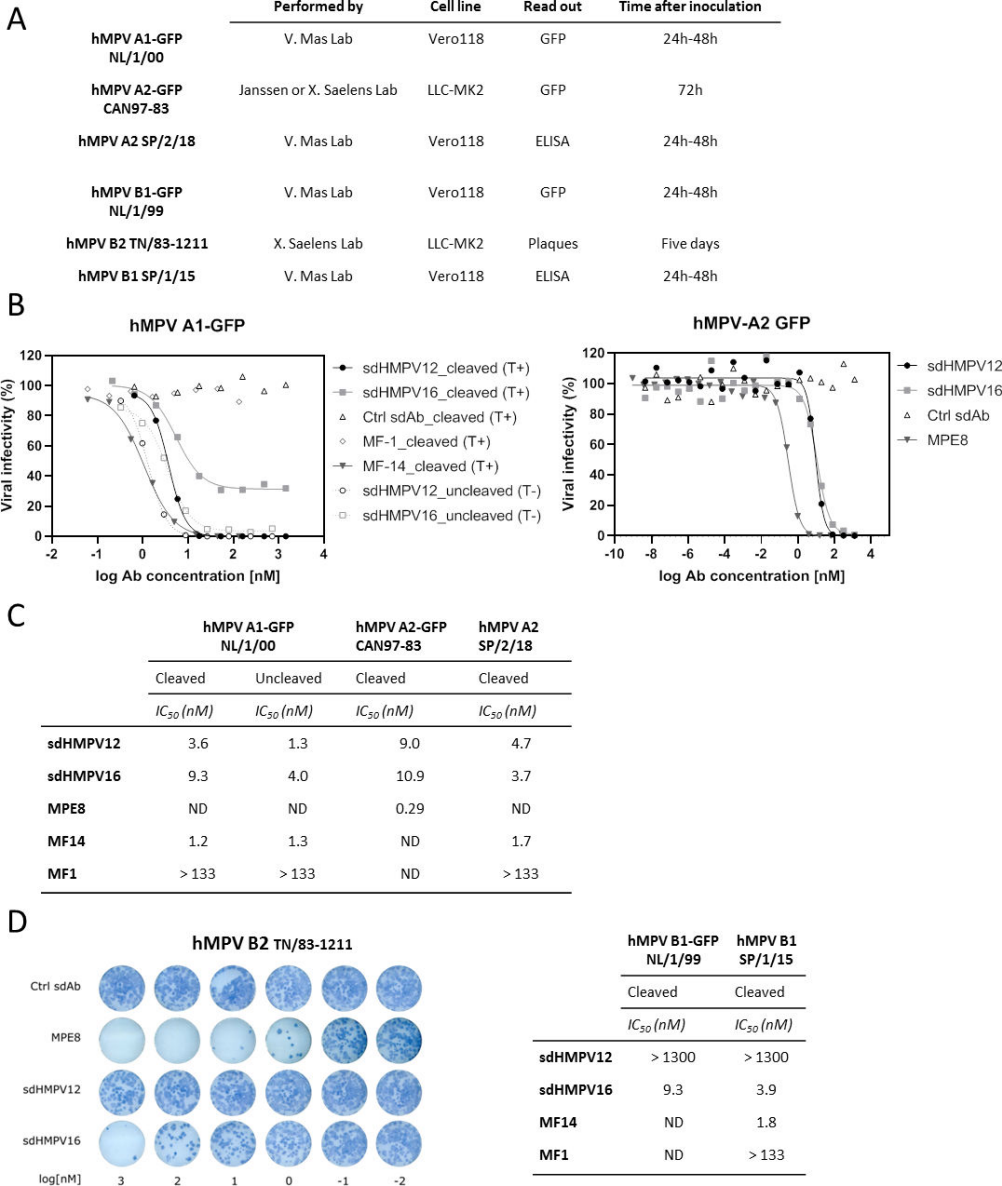
sdHMPV16 cross neutralizes hMPV A and B but sdHMPV12 only neutralizes hMPV A strains. (**A**) Details on different neutralization assay formats with sdHMPV12, sdHMPV16, or Ctrl sdAb emphasizing the different cell lines, read outs, and inoculation times. Detailed information can be found in the M&M section. (**B**) Neutralization assay results of hMPV A1-GFP isolate NL/1/00 (left panel) or hMPV A2-GFP isolate CAN97-83 (right panel). hMPV A1-GFP (NL/1/00) virus was grown in the presence (T+) or absence (T-) of trypsin. Three different mAbs were used as controls: MF-1, MF-14, and MPE8. Fluorescence intensities are expressed as a percentage of a virus control without sdAb (% viral infectivity). (**C**) Overview of the IC50 values of the neutralization assays with hMPV A strains calculated from GFP or ELISA read outs for GFP or wild-type viruses, respectively. ND = not determined. (**D**) Neutralization assay results of hMPV B2 isolate TN/83–1211 (left), hMPV B1-GFP isolate NL/1/99, and hMPV B1 isolate SP/1/15 (table on the right). hMPV B2 (TN/83–1211) plaques were stained with mouse anti-hMPV serum and not quantified. IC_50_ values for hMPV B1-GFP (NL/1/99) and hMPV B1 (SP/1/15) were calculated based on GFP or ELISA read outs.

After the initial screen, sdAbs that showed hMPV neutralizing capacity were further characterized in additional neutralization screens. It is important to note that depending on the viral strain, these assays have a different read out and inoculation time (Fig. 2A). Therefore, the neutralization activity of different mAbs or sdAbs was only compared within one type of assay for one specific viral strain or isolate. As controls, we included a sdAb directed against influenza A matrix protein 2 ectodomain (31) as well as three different monoclonal antibodies: MF1 which specifically binds hMPV Post-F and lacks neutralizing activity (11), MF14 which binds a neutralizing epitope that is conserved in hMPV Pre- and Post-F (11), and the human monoclonal antibody MPE8 which can neutralize hRSV, hMPV, bovine RSV, and pneumovirus of the mouse (32). Two sdAbs candidates showed an interesting profile in these neutralization screens. sdHMPV12 could neutralize hMPV A1 isolate NL/1/00- (IC_50_ of 3.6 nM) and hMPV A2-GFP isolate CAN97-83 (IC_50_ of 9 nM) reporter viruses as well as a more recent hMPV A2 (SP/2/18) strain (Fig. 2B and C). Surprisingly, sdHMPV16, did not fully neutralize hMPV A1-GFP (NL/1/00), with a plateau of approximately 35% infectivity remaining even at the highest concentrations of sdHMPV16 (IC_50_ of 9.3 nM; Fig. 2B). Trypsin is used in the standard protocol for hMPV viral stock preparation. In the absence of trypsin, most of the F protein remains uncleaved (F_0_), whereas in its presence most of the F_0_ is converted into F_1_-F_2_ protomers (14). Cleavage of F_0_ can occur at the cell surface or on the virion. This proteolytic cleavage likely exposes different antigenic sites in Pre-F compared with F_0_ and also increases the thermostability of Pre-F (33). One possible explanation for the incomplete neutralization of hMPV A1-GFP (NL/01/00) by sdHMPV16 could be that it targets a site that is less accessible in the cleaved Pre-F protein. Therefore, we prepared hMPV A1-GFP (NL/1/00) viral stocks in the presence and absence of trypsin. As expected, in the presence of trypsin, the F protein in the hMPV virion preparation is mostly cleaved, whereas in the absence of trypsin, most of the F protein is uncleaved (F_0_) (Fig. S1). Unlike other hMPV strains, virus preparations of hMPV A1-GFP (NL/1/00) with mainly uncleaved F protein on the virion are infectious as this recombinant virus contains the S101P substitution at the cleavage site that makes the virus less trypsin-dependent (34). sdHMPV16 can fully neutralize hMPV A1-GFP (NL/1/00) with most of the F in the F_0_ conformation (sdHMPV16_uncleaved (T-) in Fig. 2B). By contrast, for sdHMPV12, no significant difference in neutralization was observed between either of the hMPV A1-GFP (NL/1/00) virus preparations. Altogether, the results suggest that the preferred functional target of sdHMPV16 might be the uncleaved F protein. The neutralization potential of sdHMPV12 was hMPV A strain-specific since no neutralization of hMPV B1-GFP (NL/1/99), hMPV B1 (SP/17/15) or B2 (TN/83–1211) strains could be observed (Fig. 2D). For hMPV A2-GFP (CAN97-83), the IC_50_ of sdHMPV16 was comparable to sdHMPV12 (IC_50_ of ±10 nM) but higher than MPE8, which is bivalent (IC_50_ of 0.29 nM; Fig. 2C). In contrast to sdHMPV12, sdHMPV16 could neutralize hMPV B1-GFP (NL/1/99), hMPV B1 (SP/1/15), and B2 (TN/83–1211) strains (Fig. 2D).

We thus identified two hMPV-neutralizing sdAbs with a distinct neutralization profile. Sequence analysis of sdHMPV12 and sdHMPV16 using NanobodyBuilder2 (35) *via* the SAbPred toolbox (36) revealed sequence diversity in the CDR1, CDR2 and, especially, CDR3 (Fig. S2). Both sdAbs contain the conserved intradomain disulfide linkage between the framework regions 1 and 3. sdHMPV12 contains an additional cysteine pair at position 55 at the end of framework 2 and position 111A in its CDR3 (37). This non-canonical cysteine bridge in sdHMPV12 may contribute to the stabilization of the CDR3 loop (38, 39).

**Fig 3:**
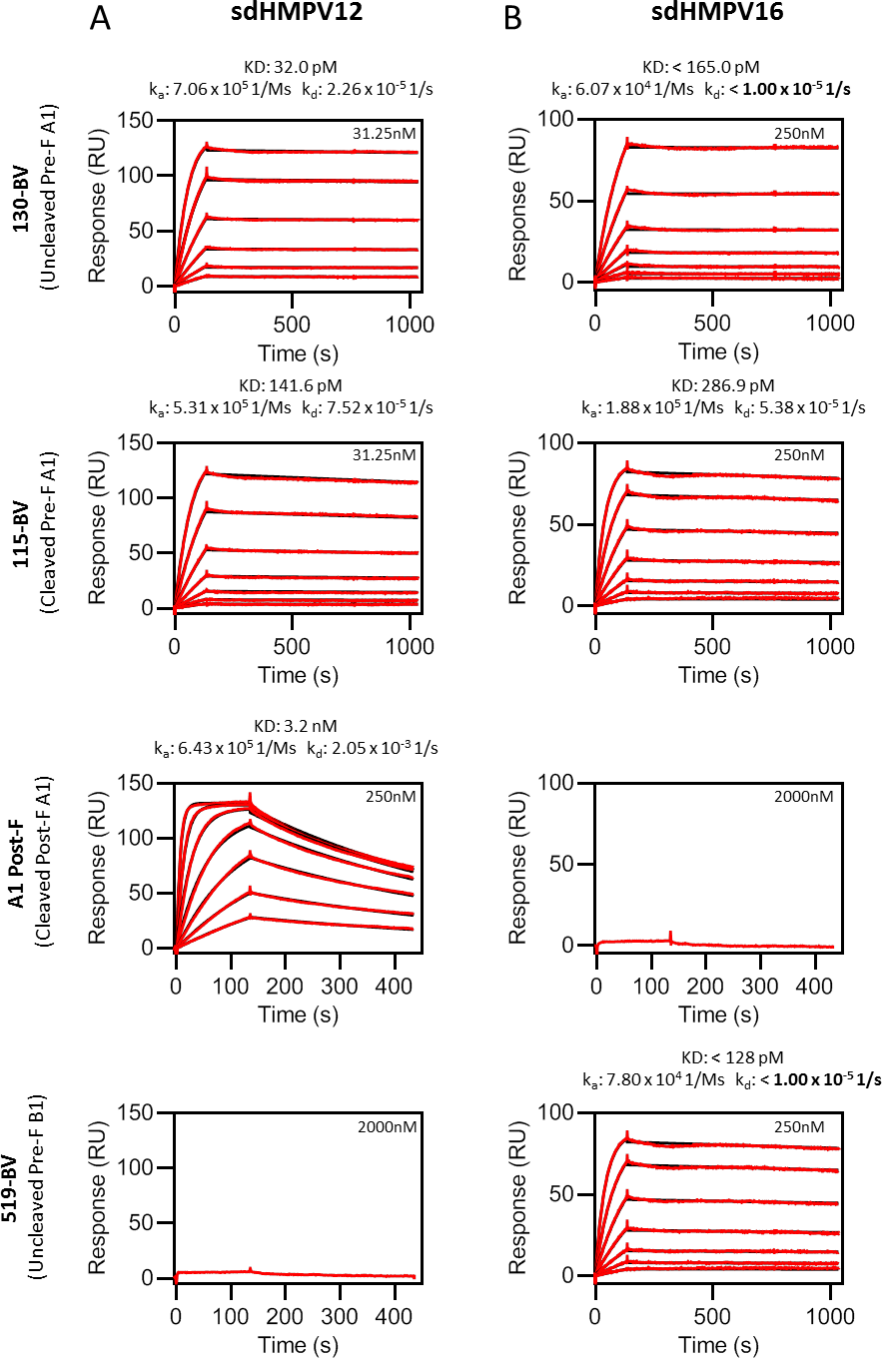
Binding of sdHMPV12 and sdHMPV16 to hMPV F variants assessed by SPR. The anti-Foldon antibody MF4chim was captured by protein A on a CM5 chip and used to capture the hMPV F variants 130-BV (uncleaved Pre-F A1), 115-BV (cleaved Pre-F A1), A1 Post-F (cleaved Post-F A1), or 519-BV (uncleaved Pre-F B1) *via* their Foldon. Serial dilutions of sdHMPV12 (**A**) or sdHMPV16 (**B**) were injected over the hMPV-F and control cells. Binding was measured with a Biacore X100 instrument as described in the Methods. Raw data and fits to the 1:1 kinetic model are shown with red and black lines, respectively. Affinity/kinetics constants and the highest nanobody concentration tested are indicated for each F protein and sdAb. The instrument detection limit for the dissociation kinetic constant (K_d_) is remarked in boldface.

To further explore the binding profiles of sdHMPV12 and sdHMPV16, their binding kinetics to recombinant Pre-F and Post-F proteins derived from hMPV A1 and B1 were determined by surface plasmon resonance (SPR) (Fig. 3). For this analysis we used a two-step F antigen capture protocol. First, the anti-foldon monoclonal antibody MF4chim was immobilized on a protein A-coated chip. Subsequently, the MF4chim was allowed to capture 130-BV by binding to the C-terminal foldon domain. sdHMPV12 bound to the uncleaved Pre-F of A1 (130-BV) with an equilibrium dissociation constant (K_D_) of 32 pM, whereas no binding with the uncleaved Pre-F of B1 (519-BV) was detected. sdHMPV12 also bound the cleaved Pre-F (115-BV) and Post-F (A1-Post) conformations of A1 with a K_d_ of 141.6 pM and 3.2 nM, respectively. These binding characteristics are in line with the observation that sdHMPV12 is a hMPV A strain-specific neutralizing sdAb and point toward a preferential tendency to bind the Pre-F conformation (Fig. 3A). sdHMPV16 bound strongly to the uncleaved and cleaved Pre-F protein preparations (A1 or B1), which is in line with the neutralization data, but unlike sdHMPV12, it did not bind to the Post-F conformation of the hMPV A1 strain indicating that this sdAb is highly Pre-F-specific (Fig. 3B). It is important to remark that sdHMPV16 complexes with uncleaved F were more stable than with the cleaved F protein. The dissociation kinetic constants for 130-BV and 519-BV could not be determined accurately even when the dissociation time was prolonged to 15 min (k_d_: <1.00 × 10^−5^ 1 /s). This different behavior in terms of binding dependency on F cleavage also appears to agree with the higher neutralization potency of sdHPMV16 against a virus stock with the majority of F in the uncleaved Pre-F conformation. In addition to affinity/kinetics determination, SPR was also used to define the antigenic sites targeted by the sdAbs. We performed competitive binding studies between sdHMPV12 and sdHMPV16 and a panel of Fabs of which the epitope has been previously reported on hMPV F. In agreement with the different F protein binding properties described above, sdHMPV12 and sdHMPV16 did not compete for the same epitope (Fig. S3A). Furthermore, based on the observed competition with Fab ADI-15614 for F binding, sdHMPV12 binds to an epitope within antigenic site III, the central region of the F protein (Fig. S3B and C).

### sdHMPV16 binds at the interface between hMPV F protomers

**Fig 4:**
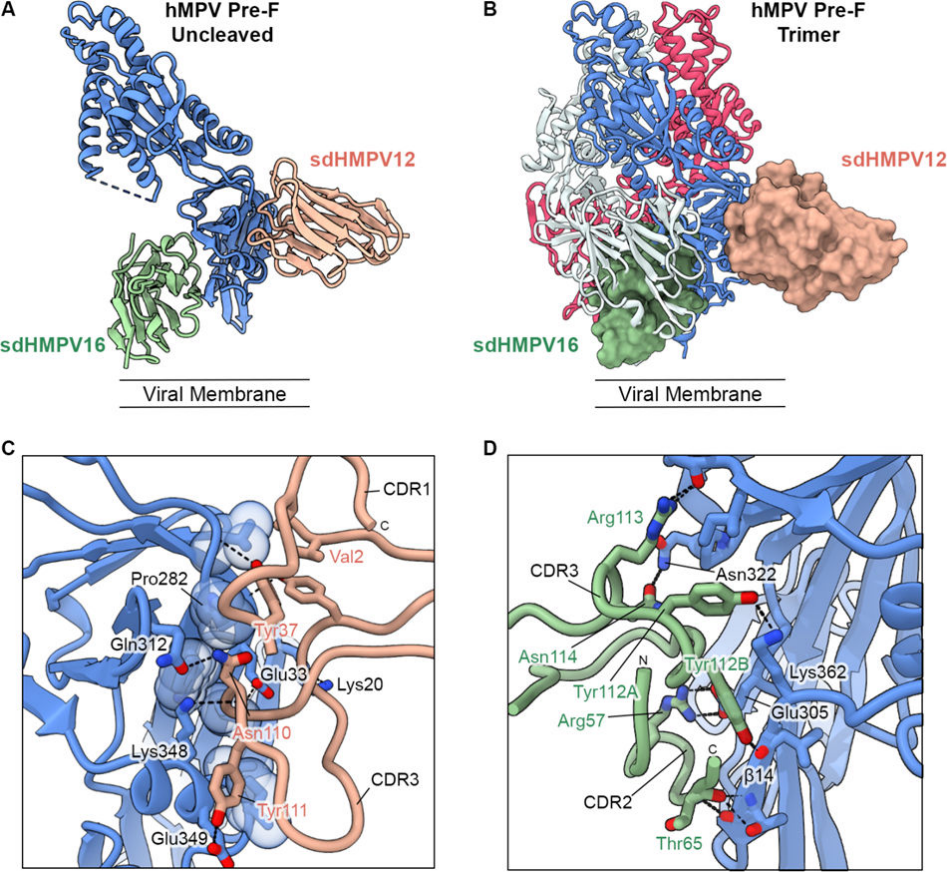
sdHMPV16 binds at the internal interface of two hMPV Pre-F protomers. (**A**) A ribbon model of sdHMPV12 (peach) and sdHMPV16 (green) bound to uncleaved monomeric hMPV Pre-F (blue) as viewed from the side. The unmodeled flexible protease cleavage site is represented by a dashed line. (**B**) Surface representations of sdHMPV12 (peach) and sdHMPV16 (green) are shown superimposed onto a ribbon model of cleaved trimeric hMPV Pre-F (PDB ID 5wb0). The sdHMPV16 surface representation is seen to clash extensively with the ribbon model of the neighboring F protomer (white). (**C**) Close-up of the sdHMPV12-binding site. The antigenic site I hydrophobic pocket residues are displayed as sticks with a transparent space-filling sphere representation of the atoms. Other residues important for the interaction are labeled and shown as sticks. CDR2 and the sdAb framework regions have been removed for clarity. (**D**) Close-up of the sdHMPV16-binding site. Important residues for the interaction are highlighted as sticks. CDR1 and the sdAb framework regions have been removed for clarity. The IMGT numbering scheme has been used to number the sdAb residues using NanobodyBuilder2 (35) *via* the SAbPred toolbox (36).

To define the epitopes recognized by sdHMPV12 and sdHMPV16, the two single-domain antibodies were complexed with uncleaved monomeric Pre-F, and a crystal structure was determined to 2.9 Å resolution (Fig. 4A; Table S1). The structure revealed that sdHMPV12 binds primarily to antigenic site I with some overlapping of antigenic site III, which explains its competition with ADI-15614 (23). The antigenic site for sdHMPV16 is located at the internal interface between protomers in the context of a trimeric hMPV F and while it has not been previously well-defined, the site was seen to be immunogenic during natural infection of humans (Fig. 4A and B) (23).

HMPV F has approximately 850 Å^2^ of its surface area buried at the sdHMPV12 binding interface. The interaction is mediated predominantly by CDR3 which packs into a hydrophobic pocket within antigenic site I while also making side chain hydrogen bond interactions with hMPV F residues Gln312 and Glu349 through Asn110 and Tyr111, respectively. Additional charged residues (Lys20, Glu33, and Lys348) at the periphery of the hydrophobic pocket also initiate contact with the CDR3 main chain through their side chains. The aromatic ring of Tyr37 in CDR1 packs against hMPV F Pro282 while also having a hydrogen bond to the hMPV F main chain *via* its hydroxyl group (Fig. 4C).

The binding of sdHMPV16 to its non-canonical antigenic site buries approximately 750 Å^2^ of surface area on uncleaved monomeric F. The interaction is mediated primarily by sdAb amino acids from CDR2 and CDR3. An important contact residue is hMPV F Glu305 which forms a salt bridge with Arg57 from CDR2. HMPV F Lys362 is positioned between two consecutive tyrosine residues from CDR3 and forms a hydrogen bond with Tyr112A while the hydrophobic portion of the Lys362 side chain packs against the aromatic ring of Tyr112B. Tyr112B further interacts *via* its hydroxyl group with the main chain of the hMPV F β14 strand. Additional β14 hydrogen bonds are formed between Thr365 and the CDR2 peptide backbone (Fig. 4D). A small hydrophobic cavity that is occupied by Phe103 from the neighboring protomer in the trimeric crystal structure is occupied here by CDR2 (Fig. S4A). Also, when comparing the sdAb structure to the previously determined trimer structure, CDR3 residues occupy the same space as the N-terminus of the fusion peptide, and this may play a part in sdHMPV16’s slight preference for uncleaved F (Fig. S4B). Furthermore, when superimposed on a single protomer of the trimer structure, sdHMPV16 clashes with the C-terminal α10 helix (Fig. S4B). This indicates that the accessibility of the sdHMPV16 epitope occurs during a dynamic Pre-to-Post intermediate state. It has previously been suggested that hMPV Pre-F may tend to exist either as a monomer or a splayed open trimer on the surface of the membrane, which would explain the ability for sdHMPV16 to bind specifically to Pre-F constructs in the SPR experiments as Post-F trimers are known to be highly stable.

A total of 4,060 partial and complete hMPV F sequences were obtained from GenBank and used to perform an F protein alignment (Fig. S5). Our crystal structure results, along with the available sequence information, elucidate the strain-specific disparities observed in neutralizing and binding experiments with both sdAbs. While the binding site of the cross-neutralizing sdHMPV16 displays a high degree of conservation, that of sdHMPV12 harbors two lineage-specific amino acid substitutions (at positions 312 and 348) directly involved in the sdAb interaction with the F protein. The combination of Gln312 and Lys348, as found in Lineage-A strains, appears to be pivotal for sdHMPV12 binding and neutralizing activity.

### sdHMPV16 binds to hMPV-infected cells and impacts F_0_ cleavage

**Fig 5:**
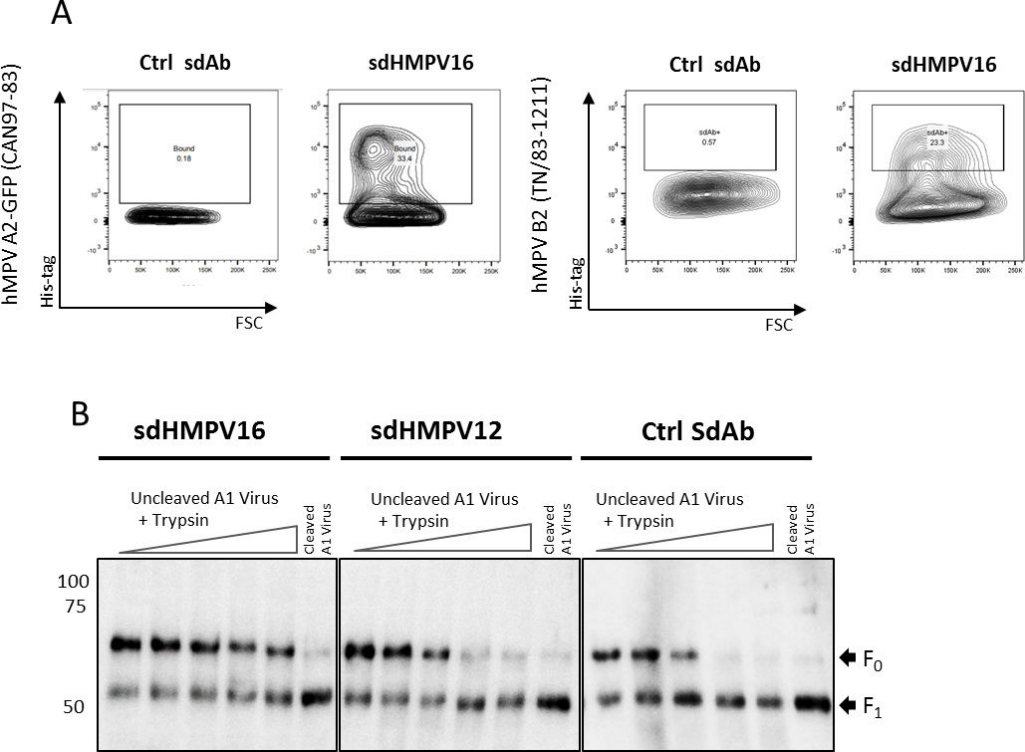
sdHMPV16 binds to infected cells and has an impact on F cleavage. (**A**) Flow cytometry contour plots showing representative staining of Ctrl sdAb (left panels) or sdHMPV16 (right panels) binding to hMPV-A2 GFP (CAN97-83) or hMPV B2 (TN/83–1211) infected cells detected with a rabbit anti-Histidine tag antibody. sdHMPV16 binds to hMPV-A2 GFP (CAN97-83) and hMPV B2 (TN/83–1211) infected cells but Ctrl sdAb does not. (**B**) Evaluation of F_0_ cleavage to F_1_ by western blot in uncleaved hMPV-A1-GFP (NL/1/00) virus preparations. Viruses were preincubated with sdAbs and then treated with different amounts of TPCK trypsin. After protease incubation samples were processed and F bands were detected using a rabbit anti-F serum. Already cleaved hMPV-A1-GFP (NL/1/00 virus preparations were also loaded onto gels as controls of almost total F_0_ cleavage). Numbers on the left correspond to molecular mass markers (kilodalton, KDa); and black arrowheads on the right indicate the position of F_0_ and F_1_ bands. A representative WB of 2 or 3 replicates for each sdAb is shown.

The unusual epitope of the hMPV-neutralizing sdHMPV16 suggests that this region of hMPV F must be partially or temporarily accessible in F on the surface of the virion envelope or hMPV-infected cells. Alternatively, the breathing of the hMPV Pre-F protein might occur, allowing sdHMPV16 to bind to and subsequently interfere with F-mediated membrane fusion. To determine whether the sdHMPV16 epitope is exposed on the surface of hMPV-infected cells, a flow cytometry experiment was performed. LLC MK2 cells were inoculated with hMPV A2-GFP (CAN97-83) and the binding of sdHMPV16 to GFP-expressing cells was determined. No binding of the irrelevant control sdAb was observed, whereas sdHMPV16 could bind to GFP-positive cells in this assay. Also, in a similar assay with hMPV B2 (TN/83–1211)-infected cells, sdHMPV16 could bind to infected cells (Fig. 5A). Next, we investigated the potential effect of sdHMPV16, sdHMPV12, and an irrelevant Ctrl sdAb on the F cleavage by trypsin. In the absence of trypsin, most of the F protein detected by western blot in the virus preparation is the F precursor (F_0_). By increasing the concentration of trypsin, more F protein is cleaved and both F_0_ and F_1_ subunits become visible. Virus pre-incubation with sdHMPV16 significantly reduced F protein susceptibility to trypsin cleavage (Fig. 5B). Unlike with sdHMPV12 or the Ctrl sdAb, in the presence of sdHMPV16, a substantial amount of F_0_ band was still detected at the highest trypsin concentration tested, indicating that sdHMPV16 hinders cleavage of F_0_ by this protease (Fig. 5B).

### sdHMPV16 fused to a human IgG1-Fc restricts hMPV replication *in vivo*

**Fig 6:**
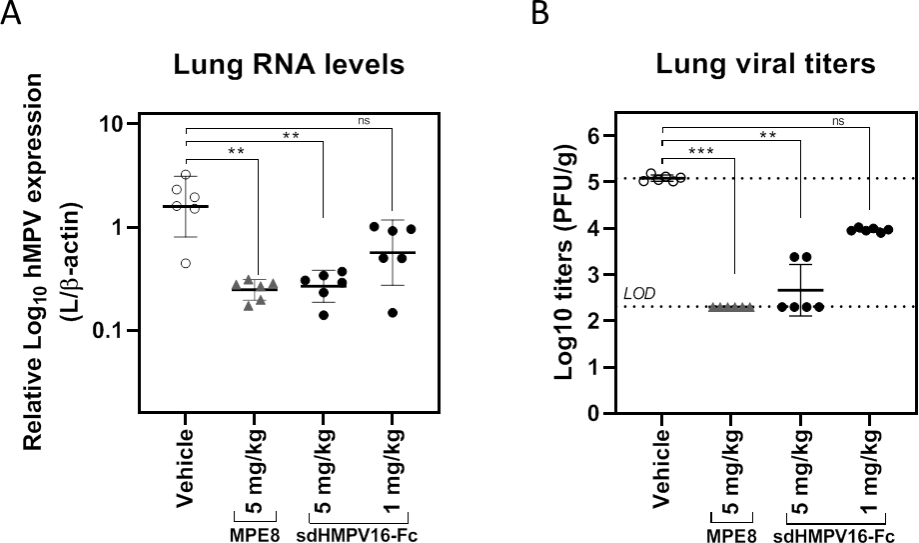
Prophylactic treatment with sdHMPV16-Fc reduces lung viral replication in cotton rats. Groups of 6 cotton rats were injected intramuscularly with vehicle (PBS), 5 mg/kg MPE8, 5 mg/kg, or 1 mg/kg sdHMPV16-Fc. The next day, the cotton rats were challenged intranasally with 10^5^ PFU of hMPV A2 isolate TN/94–49 in a 0.1 mL volume. Four days after the challenge, the pulmonary viral load was determined by quantification of the lung viral RNA (L gene) compared to β-actin mRNA using RT-qPCR (**A**) or by quantification of viral titers (PFU/g) by plaque assay (**B**). The medians of the different groups were compared using a nonparametric Kruskal-Wallis test. ***0.0001 < *P* < .001; **0.001 < *P* < .01, and ns = not significant (*P* ≥ 0.05).

We evaluated the protective potential of sdHMPV16 in cotton rats, which are permissive for hMPV (40–42). For this, sdHMPV16 was fused to human IgG1-Fc. Such a fusion not only increases the half-life in circulation compared to free sdHMPV16 but also is compatible with Chinese hamster ovary (CHO) cell-based production and standard downstream processing trajectories in the biopharma industry (43). Fusion to an Fc was associated with a minor drop in neutralizing activity compared with sdHMPV16 (Fig. S6). Intramuscular injection of sdHMPV16-Fc or MPE8 at a dose of 5 mg/kg on the day before the hMPV A2 (TN/94–49) challenge significantly reduced viral RNA levels and viral replication in the lungs (Fig. 6A and B). At a dose of 1 mg/kg of sdHMPV16-Fc, a reduction in the lung viral RNA and infectious hMPV was still observed, although this reduction did not reach statistical significance.

## DISCUSSION

Our aim was to isolate sdAbs that can neutralize hMPV A and B strains with high potency. Such sdAbs could be further developed into an antiviral product to prevent or treat diseases caused by hMPV infection. We describe a sdAb that can neutralize both hMPV A and B strains, is specific for the Pre-F conformation of hMPV F, and targets the trimer interface. sdHMPV16 was selected from a sdAb library constructed from PBMCs isolated from a llama that had been immunized with the 130-BV F antigen (11). This engineered antigen contains the stabilizing S185P substitution and the natural cleavage site (RQSR), features that allow the F to adopt a trimeric uncleaved Pre-F conformation, a potentially more open or breathable protein form that can elicit antibodies targeting external and internal surfaces of the hMPV F protein. Naturally occurring antibodies that bind to the trimeric interface have been isolated from healthy human subjects. Taking into account that the majority of individuals are seropositive for hMPV infection, these studies support the likelihood that sdHMPV16-like antibodies can also be raised after a natural hMPV infection (22, 33).

Multiple screening strategies using the same sdAb library that resulted in the isolation of sdHMPV16 led to the discovery of sdAbs that competed with sdHMPV16 for F binding, including sdHMPV15 (Fig. S7). This confirms that the 130-BV immunogen preparation partially or temporarily was present in an open trimeric state. It is, however, unclear whether the trimeric conformation was already altered at the time of immunization making it difficult to evoke a humoral response targeting hMPV F trimers, or if the closed trimer conformation of 130-BV is partially lost during bio-panning, creating a bias toward sdAbs that recognize an epitope that is located at the trimer interface, close to the foldon domain.

Recently, DS-CavEs2, a more advanced version of the hMPV F antigen was described, with a 10-fold higher expression level than 130-BV and which adopts a trimeric conformation (18). Surprisingly, DS-CavEs2 crystallized as a monomer even when complexed with MPE8 (32), which targets an epitope that spans two adjacent protomers. Nevertheless, trimeric DS-CavEs2 particles could be visualized by negative stain electron microscopy when bound by MPE8. It is thus likely that hMPV F trimers and monomers are in equilibrium even when a trimerization motif is added (18). To allow the binding of mAbs or sdAbs within the trimer, the protomers do not need to dissociate completely. Instead, they can also undergo trimer opening or so-called breathing. This phenomenon has been described for several class I viral fusion proteins such as RSV F, SARS-CoV2 spike, or the hemagglutinin of influenza A viruses (44–46). Such an open trimer configuration was also proposed to be important for integrin binding during the entry of hMPV. The hMPV RGD motif that is responsible for integrin binding is located at the F trimer interface and needs to be accessible at some point to allow entry (11). We also report that prophylactic treatment with sdHMPV16-Fc could reduce hMPV RNA levels and replication *in vivo*. To our knowledge, our cotton rat study is the first to report the therapeutic potential of targeting the intratrimeric epitope of hMPV F in an *in vivo* model.

Our data indicate that sdHMPV16 impacts the F precursor priming, a novel mechanism that has not been proposed previously for any other isolated anti-F antibody that neutralizes hMPV. This finding raises the question of the potential synergistic effects in terms of sdHMPV16-like antibodies with poor neutralization potency and other conventional neutralizing antibodies, the ones targeting the apex region of Pre-F, whose proposed mechanism of neutralization is the hijacking of Pre-F during the fusion process. To answer this question, more studies should be carried out on the beneficial effects in terms of neutralization of mixtures of antibodies. A proper F priming by cellular proteases is highly dependent on a restricted sequence. This fact would make it appropriate to develop treatments based on sdHMPV16-like antibodies since the selection of resistances would likely be strongly compromised. In addition to its impact on F cleavage by selective sampling of the open conformation, sdHMPV16 could disturb the dynamic equilibrium between open and closed hMPV F trimers, which is in line with the observation that sdHMPV16 also neutralizes virions, albeit not to completion, that mainly contain cleaved F proteins. It is possible that the opened-up Pre-F trimer conformation is an essential intermediate step during the fusion process. By binding such a conformation, sdHMPV16 could potentially slow down or prevent fusion. In immunocompromised patients, hMPV infection is often persistent and can result in severe disease (47). Therefore, it would also be of interest to, in the future, evaluate the protective potential of sdHMPV16-Fc against hMPV infection in an immunosuppressed cotton rat model (48).

sdHMPV16 targets an internal epitope and binds to Pre-F with a preference for the uncleaved conformation. The fact that sdHMPV16 can bind so deep within the F trimer supports the hypothesis that the uncleaved Pre-F trimer might adopt a more dynamic conformation which exposes some epitopes that are less accessible when the Pre-F protein is cleaved (20, 33). Stabilized Pre-F proteins of hRSV or parainfluenza viruses are known to elicit higher neutralizing antibody titers than Post-F antigens in animals and humans (49–52). Although early reports did not show an apparent advantage of immunization with Pre-F over Post-F (11, 19), recent evidence shows that the Pre-F hMPV conformation is a superior vaccine antigen that can induce higher neutralizing antibody titers than Post-F and a robust hMPV A and B cross-reactive antibody response (18). It is important to note that early Post-F protein preparations likely contained some F protein in the Pre-F conformation which might explain the discrepancy in results. Here, the 130-BV antigen was used for a llama immunization to elicit a broad range of potent hMPV Pre-F specific sdAbs. Our results suggest that the use of an uncleaved Pre-F facilitates the production of antibodies that target the internal surface of the F protein. Recently, Huang et al. reported on the monoclonal antibody MPV458 that binds at the interface between two F protomers. The MPV458 epitope is a single alpha helix of amino acids 66–87 of the F_2_ region more distal from the base of the protein compared to the sdHMPV16 epitope. Interestingly, this helix is structurally conserved in the Pre-F and Post-F conformations but, in the latter, the helix is exposed on the outer surface of the trimer (22). Since the discovery of this epitope, other mAbs that target an epitope positioned in the trimerization interface have been described (20, 23, 33).

sdAbs and sdAb-based formats are promising antiviral drug candidates. Several recent publications have described mAbs binding epitopes on the trimer interface, including antibodies binding near the membrane-proximal base of the protein, though with low neutralization potency (23). Due to its small size and the increasing amount of data supporting the breathing of the F trimer, we believe sdHMPV16 can penetrate the F trimer and sit close to the base of the trimer preventing F cleavage and hMPV fusion.

## MATERIALS AND METHODS

### Isolation of hMPV F-specific sdAbs

Immunizations and VHH library generation were performed by the VIB Nanobody Core facility according to directive 2010/63/EU of the European Parliament for the protection of animals used for scientific purposes and approved by the Ethical Committee for Animal Experiments of the Vrije Universiteit Brussel (permit No. 13–601-1). Briefly, a llama was subcutaneously immunized six times at weekly intervals with 150 µg of hMPV prefusion F protein (130-BV) in the presence of Gerbu LQ#3000 adjuvant (11). Five days after the last immunization, blood was collected and total RNA was extracted out of isolated lymphocytes. After first-strand cDNA synthesis with an oligodT primer, VHH encoding sequences were amplified and cloned into the PstI and NotI sites of a phagemid pMECS vector. In this pMECS vector, the VHH coding sequence is double tagged with an HA and 6xHis tag (AAAYPYDVPDYGSHHHHHH). Next, Electro-competent E.coli TG1 cells were transformed with the recombinant pMECS vector resulting in a VHH library. A library of VHH-presenting phages was obtained after inoculation with VCS M13 helper phages. This library was subjected to three rounds of panning on hMPV prefusion F protein (20 µg 130-BV) which was captured with an anti-foldon antibody (100 ng of MF4chim) to favor an upright orientation of the immunogen (11). Three different buffers were used in each round to block the 130-BV coated and uncoated well. SEA BLOCK blocking buffer (Thermo Scientific) for Round 1, Pierce Blocking buffer for Round 2, and 4% BSA for Round 3. After blocking, 1 × 10^12^ phage particles were incubated for 1 h at room temperature. After thorough washing, retained phages were eluted by adding a TEA solution (14% triethylamine; Sigma; pH 10) for 10 min. Dissociated phages were transferred to 1 M Tris-HCl pH 7.4 for neutralization. These phages were used to infect TG1 cells to start a new panning round.

### Periplasmic ELISA screen to identify hMPV F-specific sdAbs

After each round of panning, individual colonies were randomly selected to determine the binding properties of the sdAbs to different hMPV F proteins *via* ELISA. To prepare periplasmic extracts (PE), colonies were used to inoculate 2 mL of terrific broth (TB) medium with 100 mg/mL ampicillin. After a 5-h incubation step at 37°C, sdAb expression was induced by adding 1 mM isopropyl b-D-1-thiogalactopyranoside (IPTG). The next day, bacterial cells were pelleted and resuspended in 200 mL TES buffer (0.2 M Tris-HCl pH 8, 0.5 mM EDTA, 0.5 M sucrose). After incubation at 4°C for 30 min, 300 µL water was added to induce osmotic shock. The supernatants (=PE) were collected after an additional incubation step of 1 h at 4°C. For the ELISA, Maxisorp plates were coated overnight with a murine anti-foldon antibody (100 ng of MF4 (11)) or bovine serum albumin (BSA, Sigma-Aldrich). The coated plates were blocked with 4% milk powder in phosphate-buffered saline (PBS). Next, 100 ng 130-BV F protein was added. After washing, 50 µL of the PE was added to the wells. Bound sdAbs were detected with rabbit anti-HA (1/2,000; ab9110; Abcam) mAb and horseradish peroxidase (HRP)-linked donkey anti-rabbit IgG (1/2,000; NA934; GE Healthcare). After washing, 50 µL of TMB substrate (Tetramethylbenzidine, BD OptEIA) was added to every well. The reaction was stopped by the addition of 50 mL of 1M H_2_SO_4_, after which the absorbance at 450 nM was measured with an iMark Microplate Absorbance Reader (Bio-Rad). All periplasmic fractions for which the OD_450_ values obtained for 130-BV were at least two times higher than the OD_450_ values obtained for BSA were retained. Corresponding colonies were grown in LB medium with 100 µg/mL ampicillin for plasmid isolation using the Wizard (Promega). The DNA sequence of the VHH was determined by Sanger sequencing using the primer MP057 (5′-TTA TGC TTC CGG CTC GTA TG-3′).

### Expression of sdAbs in *Pichia pastoris*

VHH sequences were cloned into a yeast expression vector as follows. VHH sequences were PCR amplified for the pMECS plasmid using the following forward and reverse primers (5′-GGCGGGTATCTCTCGAGAAAAGGCAGGTGCAGCTGCAGGAGTCTGGG-3′;5′-CTAACTAGTCTAGTGATGGTGATGGTGGTGGCTGGAGACG GTGACCTGG-3′). The PCR products were digested with XhoI (Promega) and SpeI (Promega) and ligated into XhoI/SpeI-digested pkai61 backbone with Zeocin resistance marker (53) using T4 DNA ligase (Thermoscientific). The VHH sequences are cloned in-frame with a slightly modified version of the *S. cerevisiae* α-mating factor secretion signal. The encoded genes contain a C-terminal 6XHis tag and are under the control of the methanol-inducible AOX1 promoter. The vectors were linearized with PmeI (Thermoscientific) and transformed in the *P. pastoris* strain GS115 using the condensed transformation protocol described by Lin-Cereghino et al. (54). After transformation, the yeast cells were plated on YPD plates [1% (wt/vol) yeast extract, 2% (wt/vol) peptone, 2% (wt/vol) dextrose, and 2% (wt/vol) agar] supplemented with Zeocin (100 µg/mL; Life Technologies) for selection.

### Purification of sdAbs produced by *Pichia pastoris*

Large-scale production of sdHMPV12 and sdHMPV16 by *Pichia pastoris* was performed. On day 1, individual *P. pastoris* transformants were used to inoculate YPNG medium (2% peptone, 1% Bacto yeast extract, 1.34% YNB, 0.1 M potassium phosphate pH 6, 1% glycerol) with 100 µg/mL Zeocin (Life Technologies) and incubated while shaking at 28°C for 24 h. Next, cells were pelleted and the YPNG medium was replaced by YPNM medium (2% peptone, 1% Bacto yeast extract, 1.34% YNB, 0.1 M potassium phosphate pH 6, 1% methanol) to induce sdAb expression. Cultures were incubated at 28°C while shaking for 48 h. 1.25% methanol was added to the cultures at 16, 24, and 40 h. After 48 h, the yeast cells were pelleted and the supernatant was retained to assess the presence of sdAbs. The cleared supernatant was subjected to ammonium sulfate precipitation (80% saturation) for 4 h at 4°C. The insoluble fraction was pelleted by centrifugation at 20,000 × *g* and resuspended in 10 mL binding buffer (20 mM NaH2PO4 pH 7.5, 0.5M NaCl, and 20 mM imidazole pH 7.4). The sdAbs were purified from the solution using a 1 mL HisTrap HP column (GE Healthcare). Bound VHHs were eluted with a linear imidazole gradient starting from 20 mM and ending at 500 mM imidazole. VHH-containing fractions were pooled and concentrated with a Vivaspin column (5 kDa cutoff, GE Healthcare) and further purified by gel filtration (Superdex 200 10/300 Gl) in PBS buffer. Fractions containing sdAb were again pooled and concentrated. Purity was evaluated by SDS-PAGE followed by Coomassie blue staining.

### Cells and viruses

The Vero-118 cell line (kind gift of R. Fouchier; Erasmus Medical Center; Amsterdam; NL) was grown in Iscove’s Modified Dulbecco’s Medium (IMDM), whereas LLC-MK2 cells (ATCC CCL7), Caco2 cells, and CV-1 (CCL-70TM) cells were grown in Dulbecco’s modified eagle medium (DMEM). Both media were supplemented with 10% heat-inactivated fetal calf serum (FCS), 2 mM L-glutamine, non-essential amino acids (Invitrogen, Carlsbad, CA, USA), penicillin (100 IU/mL), streptomycin (100 µg/mL), and 1 mM sodium pyruvate at 37°C in the presence of 5% carbon dioxide. hMPV A2 -GFP (CAN97-83) strain (M121-; ViraTree; USA), hMPV B2 (TN/83–1211) strain (NR-22227; BeiResources; USA), hMPV A1-GFP (NL/1/00), and hMPV B1-GFP (NL/1/99) strain (both strains were a kind gift of B. van den Hoogen and R. Fouchier; Erasmus Medical Centre; Rotterdam; NL) were propagated on Vero118 cells in the presence of 3.75 µg/mL TPCK-trypsin (Sigma Aldrich) for 5 days. Two days before virus harvest, the infection medium (IMDM supplemented with 2% FCS, 2 mM L-glutamine, non-essential amino acids, penicillin [100 IU/mL], streptomycin [100 µg/mL], and 1 mM sodium pyruvate) was replaced with fresh medium with or without TPCK-trypsin to produce virus stocks with cleaved and uncleaved F proteins, respectively. hMPV A2 SP/2/18 (Genbank ID: OR766348) and hMPV B1 SP/1/15 (Genbank ID: OR766347) viruses were isolated after five passages in Caco-2 cells from HMPV-positive nasopharyngeal aspirate samples collected in Spain. The samples were obtained from young children with mild respiratory symptoms in the years 2015 (B1 SP/1/15) and 2018 (A2 SP/2/18). These two viruses were grown in DMEM (Gibco) supplemented with 2% FCS without trypsin for 5 days with an infection medium replacement 2 days before virus harvest. hMPV A2-GFP (CAN97-83) and hMPV B2 (TN/83–1211) stocks were quantified on LLC MK2 cells by plaque assay using in-house serum of 130-BV immunized mice (mouse hMPV serum). hMPV A1-GFP (NL/1/00, passage 5), hMPV B1-GFP (NL/1/99, passage 5), hMPV A2 (SP/2/18, passage 5), and hMPV B1 (SP/1/15, passage 5) were quantified in Vero-118 cells by foci forming units (ffu) assays after an infection prolonged for 2 days without trypsin and a subsequent F antigen detection by ELISA using a cocktail of mouse anti-F antibodies (MF1, MF14, and MF16).

### Antibodies and Fab preparations

All anti-F monoclonal antibodies were produced and purified in-house. Mouse antibodies MF1, MF14, MF16, and 101F (11, 17) were obtained from hybridoma supernatants, grown in ClonaCell HT medium (Stem Cell Technologies, cat #03805). Human antibodies MPE8, ADI-15614, and MFP4 were purified from supernatants of 293 F cells (Invitrogen) transiently co-transfected with plasmids encoding antibody heavy and light chains. The VRC-8400 plasmid backbone encoding the heavy and light chains for MPE8, ADI-15614, was obtained from the Vaccine Research Center (VRC) at the National Institutes of Health (NIH). MFP4 antibody was cloned in the same expression vector after sequencing the variable regions of a mouse hybridoma targeting F hMPV antigenic site ϕ. All the antibodies were purified from supernatants by affinity chromatography over a protein A-Sepharose CL-4B column (Cytiva #17–0780-01) as recommended by the manufacturer. For Fab preparations, purified antibodies were digested with papain (Sigma Aldrich) and the resulting mouse and human Fabs were purified as recommended by the manufacturer using a CaptureSelect LC-Kappa mur (Thermo Scientific #191315005) and a CaptureSelect Ig-CH1 (Thermo Scientific #194320005) affinity matrix, respectively.

### Cleavage of the F protein in viral stocks by Western-Blot

Ten to four (10^4^) ffu of virus stocks were loaded on a 10% SDS-PAGE gel and transferred to Immobilon membranes (Millipore) for the detection of F_0_ and F_1_ subunits using in-house serum of A1-Post-F immunized rabbit. Bands were visualized using an Amersham ECL Advance Western Blotting Detection Kit (Cytiva) and imaged using a Kodak Gel Logic 1500 Imaging System camera and Kodak Molecular Imaging software. To check sdAb’s impact on F cleavage, the viruses were pre-incubated with 0.5 mg of sdAbs for 1 h at RT. Then, samples were digested with different amounts of TPCK-trypsin (0.06, 0.2, 0.8, or 1.5 ng) for 1 h at 37°C and loaded for WB analysis as described above. The RSV anti-F nanobody VHH-L66 (25) was used as control sdAb.

### hMPV neutralization assays

Neutralization assay protocols differed depending on the viral strain and the laboratory where the assay was performed. In the V. Mas lab, neutralization assays with hMPV A1-GFP isolate NL/1/00, hMPV A2 isolate SP/2/18, and hMPV B1 isolate SP/1/15 were performed according to previous works (11, 20). Briefly, a predetermined amount of recombinant hMPV A1-GFP (NL/1/00) or wild-type viruses strains hMPV A2 (SP/2/18) or hMPV B1 (SP/1/15) were mixed with serial dilutions of purified sdAbs or mouse sera before being added to cultures of Vero-118 cells. For the GFP virus, the medium was replaced by PBS after 24–48 h, and GFP fluorescence was measured in a Tecan microplate reader M200. Values were expressed as percent of a virus control without antibody (% viral infectivity) and IC_50_ values were determined. Cell monolayers inoculated with wild-type viruses were fixed with 4% formaldehyde in PBS with Ca2+/Mg2+ and processed to F antigen detection by ELISA using a mouse anti-F antibody cocktail (MF14, MF16, and MF1). Optical density was read at 492  nm and IC_50_ values were determined. Neutralization assays with hMPV A2-GFP isolate CAN97-83 were performed at Janssen or in the X. Saelens lab. A dilution series of the sdAb/mAbs/mouse sera was prepared in Opti-MEM (Gibco) and incubated with a predetermined amount of hMPV A2-GFP (CAN97-82) at 37°C. After 1 h, LLC MK2 cells (in Opti-MEM supplemented with 1 µg/mL TPCK- Trypsin) were added and infection was allowed for 72 h after which medium was replaced by PBS and GFP fluorescence was measured in a Tecan microplate reader M200. Values were expressed as percent of a virus control without sdAb and IC_50_ values were determined. Plaque reduction assays with hMPV B2 isolate TN/83–1211 were performed in the X. Saelens lab. A dilution series of the sdAb/mAbs/mouse sera was prepared in Opti-MEM (Gibco), incubated with hMPV B2 (TN/83–1211) for 1 h at 4°C, and used to infect confluent LLC MK2 cells. After 2 h, the inoculum was removed and 0.3% avicel RC-851 Q (FMC Biopolymers) in Opt-MEM medium with 1 µg/mL TPCK-trypsin was added and the infection was allowed to continue at 37°C for 5 days. Viral infection was determined by immunostaining of the viral plaques with mouse anti-hMPV serum (in-house) and horseradish peroxidase-conjugated anti-mouse IgG (1/2,000, NXA931, GE Healthcare). The plaques were visualized by adding TrueBlue peroxidase substrate (KPL, Gaithersburg).

### Surface plasmon resonance

All the SPR experiments were carried out in a Biacore X100 instrument using captured F protein as ligands and sdAb and Fabs as analytes. Briefly, F proteins were immobilized in Protein A sensor chips (Cytiva) where the anti-foldon human recombinant monoclonal antibody MF4 was previously coupled. The first immobilization of MF4 antibody was carried out at reference and sample cells at ~4,000 response units (RU). The second immobilization of protein F was performed at a sample cell at a level of 400–500 RU. For evaluation of sdAb binding and affinity/kinetics to F constructs, a multicycle format assay was performed by injecting a range of twofold serial sdAb dilutions (5–6 sdAb concentrations) at a flow rate of 40 µL/min. The association phase was prolonged for 135 s, whereas the dissociation phase was extended up to 900 s depending on sdAb-F complexes stability. Sensorgram data were fit to a 1:1 Langmuir binding model for the calculation of the kinetic parameters ka and kd. The k_d_ was then calculated as the ratio of these two rate constants (ka/kd). All the competition assays were carried out using the 130-BV construct as the ligand. To evaluate the competition between sdAbs, individual sdAb or mixtures of sbAbs were injected at saturating concentrations. sdHMPV12 was tested at 400 nM, whereas sdHMPV15 and sdHMPV16 were injected at 1,000 nM. The competition between sdAb and Fabs was carried out by sequential injections. First, the mentioned saturating concentrations were used to inject the sdAb and, after a short stabilization period, Fabs were injected at saturating concentrations that allow responses of 200–300 RU: the MFP4 Fab at 2,000 nM, whereas the MF14, MF16, ADI15614, and 101F Fabs at 250 nM. To calculate the competition level between sdAb and Fabs, a reporting point before 10 s end of Fab injection was estimated in all SPR sensorgrams. Fab response values were expressed as percent using as a control a Fab injection over a chip surface where the F protein was captured without sdAb.

### Expression and purification of hMPV F constructs

The protein isolation and characterization of all the Pre-F (130-BV and 115-BV) and Post-F (A1-Post) constructs derived from the A1 (NL/1/00 strain) have been previously described (11). The uncleaved Pre-F B1 construct was derived from B1 (NL/1/99 strain) and expressed by a recombinant vaccinia virus as described for the A1 constructs. Briefly, plasmid pRB21 (kind gift of Dr. R. Blasco of the Instituto Nacional de Investigación y Tecnología Agraria y Alimentaria (I.N.I.A.); Madrid; ES) was used to clone the hMPV F ectodomain (amino acids 1–489) stabilized in its Pre-F conformation by the introduction of a S185P substitution. The foldon trimerization domain was added at the C-terminus of the F protein ectodomain, flanked upstream by a TEV protease site and downstream by a Xa protease site, followed by a 6xHis-tag. A plasmid coding the F construct was used to generate a recombinant vaccinia virus by the method of Blasco and Moss (55). Proteins were produced by infecting CV-1 cells as described previously (11) without co-infection of furin-expressing vaccina virus. After 48-h infection time, supernatants were harvested and clarified by low-speed centrifugation. After concentration and buffer exchange, proteins were purified using the HIS-Select Nickel Affinity Gel (Millipore #P6611) columns followed by gel filtration on a HiLoad 16/600 Superdex 200  PG column (Cityva #28989335).

For monomeric A1 Pre-F (130-BV monomer), residues 1–490 were subcloned into mammalian expression vector pαH. Trailing the hMPV F ectodomain residues is a GGGS linker followed by an HRV 3C protease site, an 8xHis tag, and a Twin-Strep-tag. Gblocks for sdHMPV16 and sdHMPV12 were ordered (IDT) and cloned into the same plasmid backbone as monomeric Pre-F. The protein complex used for crystallization was expressed by transiently co-transfecting 12 × 40 mL Freestyle 293 F cells with a total of 0.1 mg Pre-F monomer, 0.075 mg sdHMPV16, and 0.075 mg sdHMPV12 plasmids using polyethylenimine (PEI). A final concentration of 5 µM kifunensine was added to the cells 3 h after transfection. After 6 days, cell cultures were combined, and protein was purified *via* Strep-Tactin Sepharose resin (IBA) from cell supernatants that had been filtered and buffer-exchanged into PBS by tangential flow filtration. The protein complex was incubated overnight at 4°C with HRV 3C and endoglycosidase H proteases. The protein complex was further purified by size-exclusion chromatography using a Superdex 200 Increase 10/300 Gl (Cytiva) in 2 mM Tris pH 8.0, 200 mM NaCl, and 0.02% NaN_3_ running buffer.

### sdAb or mAb binding to hMPV-infected cells (flow cytometry)

LLC MK2 cells were inoculated with hMPV A2-GFP (CAN97-83) or hMPV B2 (TN/83–1211) in the presence of 1 µg/mL TPCK-trypsin. Seventy-two hours after inoculation, the cells were detached, washed, and blocked in 1% BSA. Cells were stained with 1 µg/mL sdHMPV16 or Ctrl sdAb. Afterward, the cells were washed and treated with the fixation/permeabilization solution kit according to the manufacturer’s protocol (catalog no. 555028; BD). After fixation and permeabilization, cells were stained with monoclonal mouse anti-human metapneumovirus antibody (1/500, catalog no. MAB8510; Merck/Millipore). For sdAb conditions, cells were also stained with a monoclonal rabbit anti-histidine antibody (1/1,000; catalog no. PA1-983B; ThermoScientific). The binding of primary antibodies was revealed with goat anti-mouse IgG coupled to Alexa Fluor 647 (1/600; Invitrogen), donkey anti-rabbit IgG coupled to Alexa Fluor 488 (1/600; Invitrogen), and goat anti-human IgG coupled to Alexa Fluor 594 (1/600; Invitrogen), followed by analysis on an LSR Fortessa 4 laser flow cytometer (BD). Contour plots were created with the FlowJo software.

### Crystallization and X-ray diffraction data collection

The crystal for hMPV A1 Pre-F monomer in complex with sdHMPV16 and sdHMPV12 was produced by sitting-drop vapor diffusion by mixing 100  nL of the protein complex (4 mg/mL) with 100  nL of reservoir solution containing 0.1 M HEPES pH 7.0, and 18% (wt/vol) PEG 12,000 (ProPlex (56); Molecular Dimensions). For cryoprotectant, 15 µL reservoir solution and 5 µL 100% glycerol were mixed and 1 µL was added directly to the crystallization drop. The crystal was flash-frozen in liquid nitrogen. Diffraction data for a single crystal that diffracted to 2.9 Å were collected at the SBC beamline 19ID (Advanced Photon Source, Argonne National Laboratory).

### Structure determination

X-ray diffraction data were indexed and integrated in iMOSFLM (57), before being merged and scaled in AIMLESS (58). Molecular replacement was performed in PHASER (59), and the model was subjected to multiple rounds of model building and refinement in COOT (60) and PHENIX (61), respectively. Data collection and refinement statistics are presented in Table S1.

### Expression and purification of sdHMPV16-Fc fusion protein

sdHMPV16 was fused to the Fc domain (residues 104–330) of human IgG1. Plasmid DNA encoding the fusion protein was transfected into HEK293E cells. Six days post-transfection conditioned medium was harvested. The sdHMPV16-Fc fusion protein was then purified from the clarified medium using MabSelect SuRe LX-Sepharose followed by gel filtration using a Superdex 200 Increase 26/600 column equilibrated in PBS. sdHMPV16-Fc containing fractions were pooled, filter-sterilized, and subsequently analyzed by LabChip capillary electrophoresis and LAL assay.

### Prophylactic efficacy in hMPV-infected cotton rats

Inbred male Sigmodon hispidus cotton rats between 6 and 8 weeks of age (Sigmovir Biosystems, Inc., Rockville MD) were maintained and handled under veterinary supervision in accordance with the National Institutes of Health guidelines and Sigmovir Institutional Animal Care and Use Committee’s approved animal study protocol. On Day −1, four groups of cotton rats (*n* = 6 per group) received an intramuscular injection of sdHMPV16-Fc (1 or 5 mg/kg), MPE8 (5 mg/kg) or vehicle control (PBS buffer). Twenty-four hours later (Day 0), the animals were inoculated intranasally with 10^5^ pfu hMPV A2 (TN/94/49) virus in a total volume of 100 µL. Four days post-hMPV infection (Day 4), the animals were sacrificed *via* CO_2_ intoxication. Lungs were harvested to determine viral load by plaque assay and RT-qPCR as described (20). Day 4 as the time point for assessing lung viral load was selected to capture the virus at the highest titer (peak viral load), providing a robust basis for assessing treatment effects. This point was selected by CRO, Sigmovir.

### hMPV plaque assay of lung homogenates

Lung homogenates were clarified by centrifugation and diluted in EMEM. Confluent MK-2 monolayers were inoculated in duplicates with diluted homogenates in 24 well plates. After 1-h incubation at 37°C in a 5% CO2 incubator, the wells were overlaid with 0.75% methylcellulose medium. After 7 days of incubation, the overlays were removed and the cells were fixed for 1 h and air-dried for immuno-staining. Upon blocking the wells with 1% BSA in PBS, mouse anti-hMPV N protein at a1:1,000 dilution in 1% BSA was added to each well, followed by washes and then incubation with HRP-conjugated rabbit anti-mouse IgG diluted at 1:1,000 in 1% BSA. AEC Chromogen detection solution was added to each well and incubated at room temperature for 2 h. Visible plaques were counted and virus titers were expressed as plaque-forming units per gram of tissue. Viral titers were calculated as geometric mean ± standard error for all animals in a group at a given time.

### RT qPCR assay on lung homogenates

Total RNA was extracted from homogenized lung tissue using the RNeasy purification kit (QIAGEN). One μg of total RNA was used to prepare cDNA using Super Script II RT (Invitrogen) and oligo dT primer (1 µL, Invitrogen). For real-time PCRs, the Bio-Rad iQTM SYBR Green Supermix was used in a final volume of 25 µL, with final primer concentrations of 0.5 µM. Reactions were set up in duplicates in 96-well trays. Amplifications were performed on a Bio-Rad iCycler for 1 cycle of 95°C for 3 min, followed by 40 cycles of 95°C for 10 s, 60°C for 10 s, and 72°C for 15 s. The baseline cycles and cycle threshold (Ct) were calculated by the iQ5 software in the PCR Base Line Subtracted Curve Fit mode. Relative quantitation of DNA is applied to all samples. The standard curves were developed using a serially diluted cDNA sample most enriched in the transcript of interest (e.g., lungs from day 4 post-primary hMPV/A2 infection). The Ct values were plotted against the log10 cDNA dilution factor. These curves were used to convert the Ct values obtained for different samples to relative expression units. These relative expression units were then normalized to the level of b-actin mRNA (“housekeeping gene”) expressed in the corresponding sample.

## SUPPLEMENTAL MATERIAL

Supplemental material mbio.02122-23-s0001.pdf Fig. S1-S7; Table S1.

ASM does not own the copyrights to Supplemental Material that may be linked to, or accessed through, an article. The authors have granted ASM a non-exclusive, world-wide license to publish the Supplemental Material files. Please contact the corresponding author directly for reuse.
