# Supplementary material for: A neutralizing single-domain antibody that targets the trimer interface of the human metapneumovirus fusion protein
Source: mBio. 2023 Dec 20;15(1):e02122-23. doi: 10.1128/mbio.02122-23 (PMC10790764; doi:10.1128/mbio.02122-23)
Supplement: Supplemental material — Fig. S1-S7; Table S1. [file mbio.02122-23-s0001.pdf]

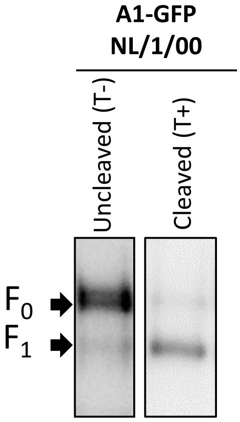

Supplementary Figure 1: Western blot detection of the F protein in hMPV A1-GFP (NL/1/00) viral stocks. Virus stocks were produced in Vero-118 cells in the absence (T-) or presence (T+) of trypsin. In the absence of trypsin most of the F protein is uncleaved (F_0) and the addition of trypsin induces cleavage and the appearance of F_1.

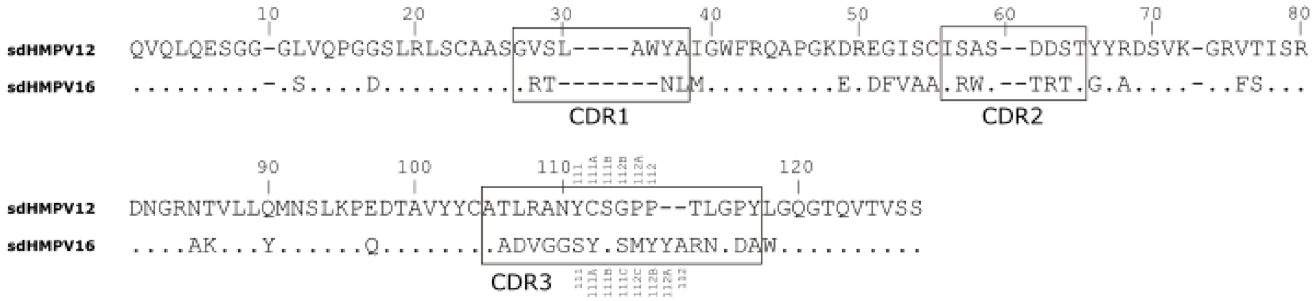

Supplementary Figure 2: Predicted amino acid sequences of sdHMPV12 and sdHMPV16 with IGMT numbering. The three complementarity-determining regions (CDR1-3) are boxed. sdAb sequences were analysed using NanobodyBuilder2 (35) via the SAbPred tool box (36).

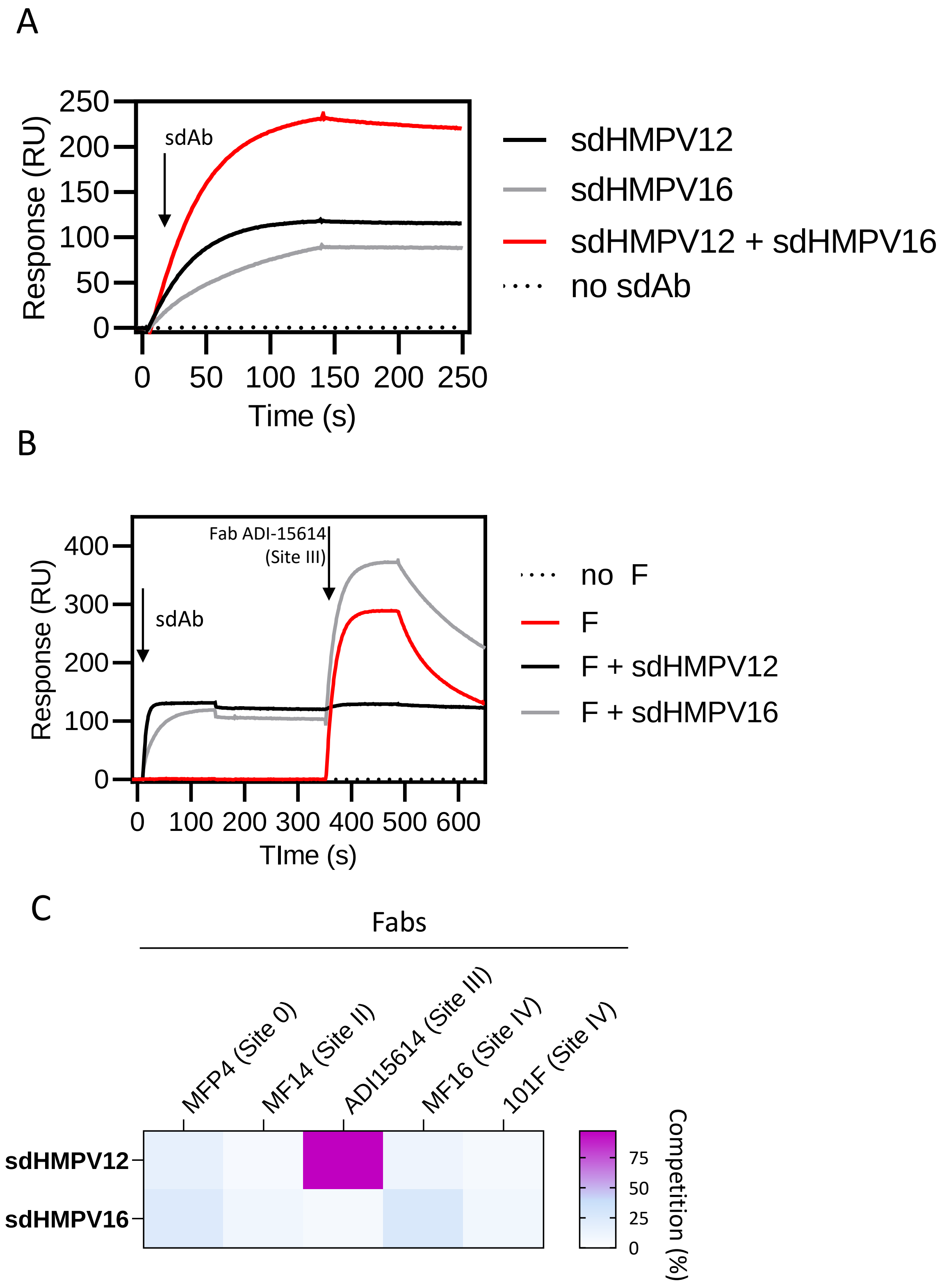

Supplementary Figure 3: Antigenic sites targeted by sdAbs. (A) SPR competition assay of binding of sdAbs to F protein. Individually sbAb or mixtures of sbAbs were injected at saturating concentrations over the captured 130-BV construct (uncleaved Pre-F A1) and control cells. The arrow indicates the sdAb injection time. An enhancement of overall response of the mixture sdHMPV12 + sdHMP16 comparing to individually sdAb injections reveals that both analytes do not compete for the same antigenic site. (**B)** Competition between sdAbs and Fabs that target the main described antigenic sites in hMPV F protein determined by SPR. sdAbs and Fabs were injected sequentially as arrows indicate to look for competition to the captured 130-BV construct. As example, a representative competition profile for Fab ADI-15614 (Site III) and two sdAbs is shown. Both types of analytes were injected at saturating concentrations. **(C)** Heat map plot of the percentage of competition achieved for each sdAb and Fab combination tested. To obtain the competition rate the report point at 480s time (B) was evaluated in each SPR trace. sdHMP12 and Fab ADI-15164 strongly compete for binding to F protein.

A

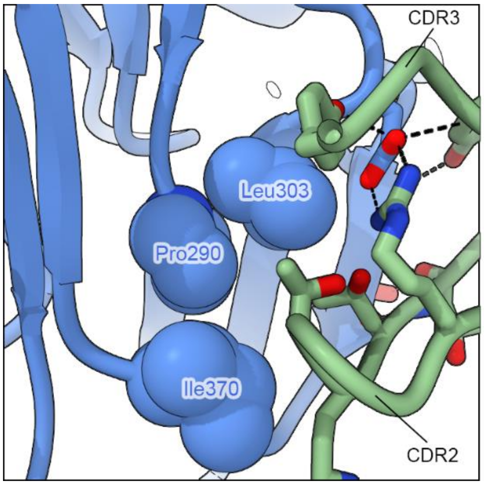

B

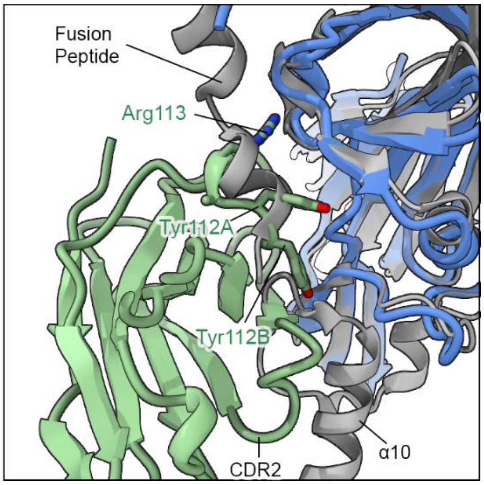

Supplementary Figure 4: sdHMPV16 binding to hMPV F necessitates the exclusion of hMPV Pre-F structural elements. (A) CDR2 and CDR3 of sdHMPV16 (green) are shown occupying the small hydrophobic cavity of the hMPV F protomer (blue) that is normally occupied by Phe103 of a neighboring protomer in trimeric hMPV Pre-F. Hydrophobic residues are shown as spherical atom representations. **(B)** A single protomer from the trimeric hMPV Pre-F crystal structure (PDB ID 5wb0; grey) is superimposed onto the sdHMPV16 structure to show would-be clashes with the fusion peptide and C-terminus of Pre-F. The IMGT numbering scheme has been used to number the CDR3 residues.

*F protein residues with side chains involved in hydrogen bond interactions with sdAb	Lineage A- specific aa substitution	Lineage B- specific aa subtitution

sdHMPV12 binding site

F2 subunit (aa)	19	20	22	24	31	33	34	35	36
	Met 799/799 (100%)	Lys* 798/800 (99.8%)	Ser 797/800 (99.6%)	Leu 799/800 (99.9%)	Ile 800/800 (100%)	Glu* 799/800 (99.9%)	Gly 800/800 (100%)	Tyr 800/800 (100%)	Leu 800/800 (100%)
		Gln 1/800 (0.1%)	Asn 2/800 (0.3%)	Ser 1/800 (0.1%)		Lys 1/800 (0.1%)			
		Arg 1/800 (0.1%)	Gly 1/800 (0.1%)						

F1 subunit (aa)	280	281	282	284	312	314	315	331
	Asp 2649/3299 (81.7%)	Thr 3297/3299 (99.9%)	Pro 3295/3299 (99.9%)	Trp 3298/3299 (99.97%)	Gln* 1764/3276 (53.8%)	Ala 3146/3147 (99.97%)	Gly 3144/3146 (99.97%)	Asp 3115/3115 (100%)
	Asn 592/3299 (17.9%)	Pro 2/3299 (0.1%)	Ser 2/3299 (0.1%)	Ile 1/3299 (0.03%)	Lys 1510/3276 (46.1%)	Thr 1/3147 (0.03%)	Arg 1/3146 (0.03%)	
	Gly 7/3299 (0.2%)		Cys 1/3299 (0.03%)		Pro 1/3276 (0.03%)		Trp 1/3146 (0.03%)	
	Glu 2/3299 (0.1%)		His 1/3299 (0.03%)		Cys 1/3276 (0.03%)			
	His 2/3299 (0.1%)							
	Lys 1/3299 (0.03%)							
	Thr 1/3299 (0.03%)							

F1 subunit (aa)	345	346	348	349	351	352
	Glu* 2784/2791 (99.75%)	Gln 2675/2679 (99.85%)	Lys* 1328/2673 (49.68%)	Glu* 2648/2653 (99.81%)	Asn 2639/2643 (99.85%)	Ile 2593/2608 (99.42%)
	Asp 6/2791 (0.21%)	His 2/2679 (0.07%)	Arg 1343/2673 (50.24%)	Asp 3/2653 (0.11%)	Asp 1/2643 (0.04%)	Thr 10/2608 (0.38%)
	Lys 1/2791 (0.04%)	Leu 1/2679 (0.04%)	Ser 1/2673 (0.04%)	Gly 1/2653 (0.04%)	Ile 1/2643 (0.04%)	Leu 2/2608 (0.08%)
		Tyr 1/2679 (0.04%)	Thr 1/2673 (0.04%)	Ser 1/2653 (0.04%)	Gln 1/2643 (0.04%)	Val 2/2608 (0.08%)
					Ser 1/2643 (0.04%)	Ser 1/2608 (0.04%)

sdHMPV16 binding site

F1 subunit (aa)	303	304	305	306	307	319	321	322	325	339	340	341	343
	Leu 3282/3282 (100%)	Arg 3266/3280 (99.6%)	Glu* 3275/3282 (99.8%)	Asp 3275/3282 (99.9%)	Gln 3277/3281 (99.9%)	Tyr 3145/3147 (99.9%)	Tyr 3146/3147 (99.9%)	Asn* 3119/3121 (99.9%)	Asp 3118/3121 (99.9%)	Asp 2961/2966 (99.8%)	Gly 2962/2965 (99.9%)	Ile 2958/2961 (99.9%)	Val 2860/2862 (99.9%)
		Lys 13/3280 (0.4%)	Lys 8/3282 (0.24%)	Glu 3/3282 (0.09%)	Lys 2/3281 (0.06%)	Phe 2/3147 (0.06%)	Leu 1/3147 (0.03%)	Asp 1/3121 (0.03%)	Ala 1/3121 (0.03%)	Thr 3/2966 (0.1%)	Arg 2/2965 (0.07%)	Phe 2/2961 (0.07%)	Val 2/2862 (0.07%)
		Gln 1/3280 (0.03%)	Asp 1/3282 (0.03%)	Asn 2/3282 (0.06%)	Pro 1/3281 (0.03%)			Phe 1/3121 (0.03%)	Glu 1/3121 (0.03%)	Pro 1/2966 (0.03%)	Trp 1/2965 (0.03%)	Thr 1/2961 (0.03%)	
			Arg 1/3282 (0.03%)	Ala 1/3282 (0.03%)	Arg 1/3281 (0.03%)				Lys 1/3121 (0.03%)	Ser 1/2966 (0.03%)			
				Ser 1/3282 (0.03%)									

F1 subunit (aa)	360	361	362	363	364	365	366	367	368	370	372
	Pro 2380/2381 (99.9%)	Cys 2356/2356 (100%)	Lys* 2354/2356 (99.9%)	Val 2184/2199 (99.3%)	Ser 2175/2190 (99.3%)	Thr* 2177/2189 (99.5%)	Gly 2174/2187 (99.4%)	Arg 2132/2135 (99.86%)	His 2098/2099 (99.9%)	Ile 2090/2090 (100%)	Met 2090/2090 (100%)
	His 1/2381 (0.04%)		Glu 1/2356 (0.04%)	Ala 14/2199 (0.64%)	Ala 8/2190 (0.37%)	Gln 8/2189 (0.37%)	Glu 8/2187 (0.37%)	Gly 3/2135 (0.14%)	Asn 1/2099 (0.05%)		
			Asn 1/2356 (0.04%)	Ile 1/2199 (0.05%)	Gly 3/2190 (0.14%)	Lys 2/2189 (0.09%)	Arg 3/2187 (0.14%)				
					Arg 2/2190 (0.09%)	Ile 1/2189 (0.05%)	Lys 1/2187 (0.05%)				
					Cys 1/2190 (0.09%)	Asn 1/2189 (0.05%)	Val 1/2187 (0.05%)				
					Thr 1/2190 (0.09%)						

Supplementary Figure 5: Sequence conservation of nanobody epitopes. Binding site for each sdAb were defined using structural information, taking into account the F protein residues within 5 Å proximity of the nanobody. A total of 4060 partial and complete hMPV F sequences were obtained from GenBank and used to perform a F protein alignment. The frequency of the amino acid substitutions in each position, grouped by F2 and F1 subunits and F protein regions, is presented.

A

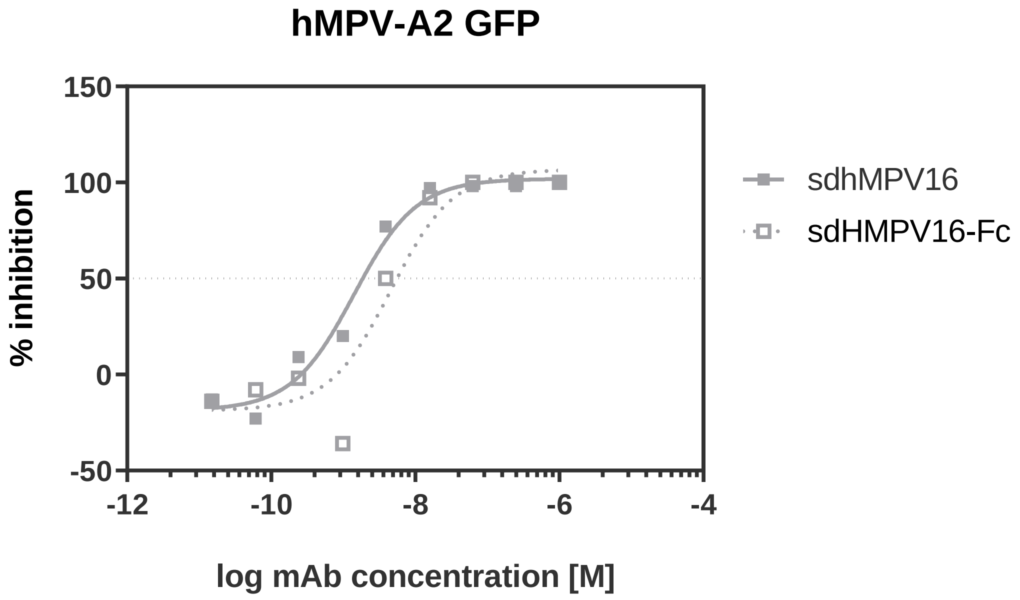

B

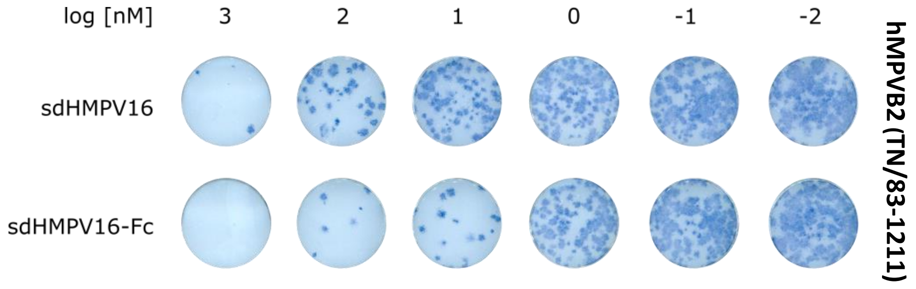

Supplementary Figure 6: Neutralization potential of sdHMPV16-Fc format. (A) hMPV A2-GFP (CAN97-83) was pre-incubated with different dilutions of sdHMPV16 or sdHMPV16-Fc before infection LLC-MK2 cells. 72 hrs later, the GFP fluorescence was measured and fluorescence intensities are expressed as percentage of a virus control without sdAb (% inhibition). **(B)** hMPV B2 (TN/83-1211) was pre-incubated with different concentrations of different dilutions of sdHMPV16 or sdHMPV16-Fc before infection of LLC-MK2 cells. After 5 days, the viral plaques were stained with mouse anti-hMPV serum. For sdHMPV16, the same results are depicted as in Figure 2C.

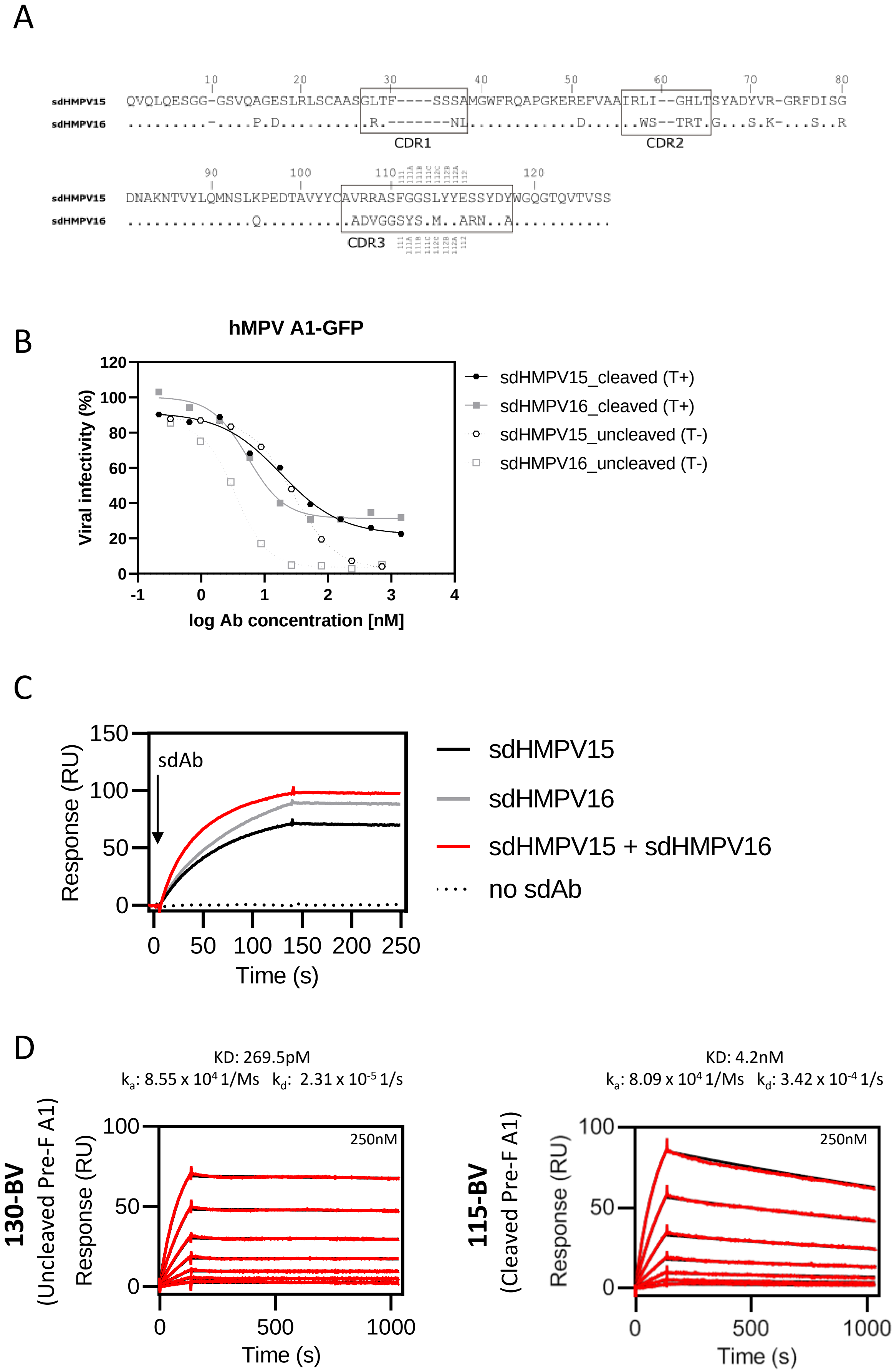

Supplementary Figure 7: Binding of sdHMPV15 to Pre-F A1 depends on F cleavage. (A) Predicted amino acid sequences of sdHMPV15 and sdHMPV16 with IGMT numbering. The three complementarity-determining regions (CDR1-3) are boxed. sdAb sequences were analysed using NanobodyBuilder2 (35) via the SAbPred tool box (36). **(B)** Neutralization of hMPV A1-GFP (NL/1/00) grown in the presence (T+) or absence (T-) of trypsin by sdHMPV15 or sdHMPV16. Data for sdHMPV16 is also shown in Figure 2A. **(C+D)** Affinity/kinetics analysis of the interaction between sdHMPV15, a strong competitor of sdHMPV16, and Pre-F A1 versions determined by SPR. Data are presented as in Figure 3.

Supplementary Table 1. Crystallographic data collection and refinement statistics.

	Uncleaved hMPV F + sdHMPV16 + sdHMPV12
**Data collection**	
Space group	$P2_12_12_1$
Cell dimensions	
a, b, c (Å)	80.3, 115.6, 158.0
α=β=γ (°)	90
Resolution (Å)	44.97-2.90 (3.00- 2.90)*
R_{merge}	0.21 (0.86)
$I / \sigma I$	7.0 (2.4)
$CC_{1/2}$	0.962 (0.638)
Completeness (%)	97.9 (99.2)
Redundancy	5.6 (5.7)
Total reflections	181,517 (27,228)
Unique reflections	32,652 (32,59)
**Refinement**	
Resolution (Å)	44.97-2.90 (3.00- 2.90)
Unique reflections	32,631 (3,255)
R_{work} / R_{free} (%)	19.85/23.95
No. atoms	9,575
Protein	9,519
Water	0
Carbohydrate	108
B-factors (Å^2)	
Protein	44.1
Carbohydrate	68.8
R.m.s. deviations	
Bond lengths (Å)	0.003
Bond angles (°)	0.50
Ramachandran (%)	
Favored	96.7
Allowed	3.3
Outliers	0.0

*Data in parentheses are for the highest resolution shell.
